# Small Is Big: Interactive Trumps Passive Information in Breaking Information Barriers and Impacting Behavioral Antecedents

**DOI:** 10.1371/journal.pone.0169326

**Published:** 2017-01-18

**Authors:** Ariane L. Beck, Kiran Lakkaraju, Varun Rai

**Affiliations:** 1 LBJ School of Public Affairs, The University of Texas at Austin, Austin, TX, United States of America; 2 Sandia National Labs, Albuquerque, United States of America; 3 Mechanical Engineering Department, The University of Texas at Austin, Austin, TX, United States of America; TNO, NETHERLANDS

## Abstract

The wealth of information available on seemingly every topic creates a considerable challenge both for information providers trying to rise above the noise and discerning individuals trying to find relevant, trustworthy information. We approach this information problem by investigating how passive versus interactive information interventions can impact the antecedents of behavior change using the context of solar energy adoption, where persistent information gaps are known to reduce market potential. We use two experiments to investigate the impact of both passive and interactive approaches to information delivery on the antecedents (attitudes, subjective norms, and perceived behavioral control in the Theory of Planned Behavior) of intentions and behavior, as well as their effect on intentions and behavior directly. The passive information randomized control trial delivered via Amazon Mechanical Turk tests the effectiveness of delivering the same content in a single message versus multiple shorter messages. The interactive information delivery uses an online (mobile and PC) trivia-style gamification platform. Both experiments use the same content and are carried out over a two-week time period. Our findings suggest that interactive, gamified information has greater impact than passive information, and that shorter multiple messages of passive information are more effective than a single passive message.

## Introduction

The increasing presence of digital media has increased the ability of consumers to easily gather an abundance of information to aid decision-making and behavior change. However, access to information alone is not sufficient, and how information is conveyed can be as important as the information itself [1,2]. Finding reliable and trusted sources of information can be challenging and time consuming to the point of overwhelming information seekers and discouraging or delaying a decision based on that information [3,4]. Accordingly, outreach efforts are now beginning to learn and understand how to leverage digital media to effectively deliver information and overcome these barriers.

An important factor is the rise of *interactive* digital media. It is now trivially easy to develop experiences that allow consumers to interact with each other, to share information, or to compete in performing certain tasks. The use of such motivational affordances is often referred to as “gamification” and can increase a user’s motivation and engagement with the information [5], thus potentially inducing behavior change. Researchers have documented several such examples, including energy conservation [6] and health related behavior change [7,8]. In contrast, passive information in this context denotes traditional, non-interactive media, such as TV Ads, newspaper ads, newsletters, flyers, posters, etc. that emphasize the passive nature of information dissemination. But few studies have examined the impact of interactive information campaigns on behavior change, especially in comparison to conventional passive information campaigns.

Both interactive and passive information campaigns have pros and cons. Interactive campaigns may engage consumers more, leading to higher impact, but come at a higher cost of creation and with potential subject sampling bias. Passive campaigns are cheaper and can reach a more diverse audience, but may not engage consumers in a meaningful or impactful way. The goal of this paper is to compare the effect of passive and interactive information campaigns on the antecedents of behavior. We address this goal using the context of solar energy adoption, where information gaps, technological advances, anchored perceptions of solar as expensive, and rapid changes in solar costs and available incentive and financing options present a wealth of dynamic information that can be challenging to navigate [4,9–13]. Additionally, the visibility of solar photovoltaic (PV) and single, unambiguous behavioral action (installed or not installed) is particularly well suited for study.

In a recent randomized control trial (RCT), dubbed “Energy Games,” we evaluated changes in attitude, subjective norms, and perceived behavioral control (PBC) toward solar in the context of the Theory of Planned Behavior [14,15] in response to a mobile, trivia-style game (discussed in more detail below). Those initial results showed a significant increase in PBC and intentions toward solar adoption, which suggested that serious games have potential for accelerating solar adoption [16]. Building on these results, in this study we completed two additional experiments to address some unresolved questions: Are the results (i.e., increase in PBC and intentions) repeatable? Will the results be similar in a different market context? Was it the game or the information that drove changes in behavioral attributes? In other words, while serious games information delivery in Rai and Beck (2016) showed encouraging results by positively impacting attributes toward adoption of solar, was it more successful than the same content delivered in a passive format? The game delivers one question at a time over a period of two weeks. Do smaller bits of information over time, as in Energy Games, have greater impact than all the information delivered at once?

To address these questions concerning interactive versus passive information we designed two new experiments, focusing on solar adoption information interventions: 1) a repeat of Energy Games using a within subjects design and a larger sample size and 2) an RCT design with participants from Amazon Mechanical Turk delivering the same content as in Energy Games but in a passive format. The within subjects design of Energy Games seeks to repeat the results of the initial Energy Games experiment [16] in a more mature solar market using a larger sample. The passive information study tests the effectiveness of delivering the same content as the game in a single passive message (single) and broken into two shorter messages (multi). As with the original Energy Games experiment in Rai & Beck (2016), both of these experiments use a pre-survey to capture demographic data, behavioral antecedents as defined by the Theory of Planned Behavior (TPB), intentions, and behavior toward solar [16]. Further, a post-survey was used to measure changes in the TPB antecedents, intentions, and behavior after the information interventions. Our findings show that the interactive game is more effective at changing behavioral antecedents than the passive information. Furthermore, the multi message condition is more effective than the single message condition compared to a control group.

## Background and Relevant Literature

The TPB model is frequently applied to understanding intentions and behavior across many disciplines, such as health, environment, education, and transportation choices [17–22]. TPB identifies three antecedents of intentions: *attitudes* toward the behavior formed from behavioral beliefs–beliefs about the likely outcomes of a behavior and the evaluations of those outcomes; *subjective norms* formed from the normative expectations of others and motivation to comply with such expectations; and *perceived behavioral control* (PBC) based on beliefs regarding factors that may enable or hinder the behavior [14,23]. The behavioral intention and PBC then directly impact behavior.

A meta-analysis of nearly 200 TPB studies by Armitage & Conner (2001) found that 39% of variance in intention and 27% of variance in behavior could be explained through TPB [17]. Bamberg and Möser’s meta-analysis specifically on pro-environmental studies also finds that 27% of variance in behavior can be accounted for by TPB, and that 53% of intentions are accounted for by PBC, attitude, and moral norm [18]. Webb *et al*. review health interventions delivered via the Internet, finding that interventions designed using TPB not only have substantial effects on behavior, but also have larger effect sizes than interventions using other behavioral models [24]. Bamberg *et al*. (2003) apply TPB in a study on travel-mode choice going beyond understanding behavior and leveraging the theory to evaluate the effectiveness of a behavioral intervention in the domain of pro-environmental behavior [25]. They find that past behavior has limited effect on future behavior if the conditions or context of the behavioral decision change, which is particularly relevant to technologies, such as solar, for which declining prices and technological advancement are consistent features of the landscape. Effectively communicating this changing context to potential adopters becomes critical to the process of reassessing decisions to adopt or reject a technology as it evolves.

To carry out the two experiments in this study, we used Energy Games, discussed below, and Amazon Mechanical Turk (AMT), an online marketplace of tasks where workers can login to the site and complete “Human Intelligence Tasks” (HITs) posted by requestors. Anyone can be a worker and/or requestor. Workers receive small payments for completing tasks, on the order of $0.10 per task. Requestors can also provide bonuses for good work. HITs vary significantly in terms of content. Some HITs are simple image classification tasks, while other HITs require evaluating/summarizing text content, or completing surveys. Once workers complete a HIT, the requestor can decide whether to accept (and pay the described amount) or reject the HIT. The number of rejected HITs is tracked for individual workers.

Several surveys of workers on AMT indicate that [26,27]: (1) workers come from many countries (>100, with the majority of workers from India and the U.S.); (2) workers have a mean age of 32, median of 30; (3) the majority of workers earn around $30K, some >$100K annually; (4) of workers who chose to give their gender, 55% were female and 45% male. These results indicate that the worker population is highly diverse, especially as compared to standard population used in university based laboratory experiments.

Importantly, requestors can require HITs to be completed by specific types of workers, which is implemented through a “qualification” system where requestors set certain qualifications workers must meet. Amazon provides system wide qualifications such as the location from where one is logging in, whether the user can view adult content, and a minimum percentage of accepted HITs. Requestors can also create custom worker qualifications.

AMT is rapidly becoming a popular method for conducting experiments due to its large and diverse subject pool, low relative cost, and the rapidity of collecting results. For instance, using AMT Paolacci et al. (2010) replicated the results of several classic psychology studies on framing effects, the conjunction fallacy, and outcome bias [26]. Experiments can be rapidly completed as with Mason & Suri's (2012) study showing that for a simple survey, at a cost of $.05 per survey response, 500 people responded within *10 days* [28]. A laboratory experiment of the same size could take months to complete and be significantly more expensive. The location qualification of AMT also allows researchers to conduct experiments specifically designed to understand the impact of culture on behavior. For instance, Eriksson and Simpson (2010) studied risk preferences as a function of gender and culture (U.S. or India)[29].

While there are many forms of interactive information [30], we chose to implement this interactive information study through “serious games”–games with a primary purpose other than entertainment–due to the ability to provide immediate feedback and a cohesive environment for a breadth of topics. Though often used interchangeably, serious games differ from “gamification” in that gamification, rather than being a self-contained game, is “the use of game design elements in non-game contexts” [31]. However, both serious games and gamification pull from a similar set of game design elements that provide motivational affordances, thus we pull from the literature on both methods.

A number of studies have investigated the effectiveness of serious games and gamification [5,32]. In a gamification survey focusing on increasing user activity (engagement) and attention, the results are slightly positive that gamification can effect motivation and increase comprehension of material [33]. However, these results can be specific to the game design and mechanics, which have many facets (e.g., motivational affordances, subject matter, game genre, audience) making generalization difficult [8].

Connolly *et al*. (2012) identify only twelve RCTs in their review of computer games and serious games studies related to learning, skill enhancement, and engagement, of which six focused on knowledge acquisition, only one focused on behavior change, and none of these addressed energy topics [32]. A 2016 update of this review applying the same methodology from 2009 to 2014 identifies an increase in the quality and number of studies related to serious games [34]. Morewedge *et al*. found that training provided with videos or games were effective at reducing bias, but that the effect size was larger for games, which was attributed to the ability to provide personalization and feedback [35]. Both videos and games showed persistence over the two month follow up period; however, it was not clear whether participants would apply this type of training to dissimilar or unfamiliar domains. A number of studies also support the effectiveness of games in health related behavior change. Baranowski *et al*. targeted increasing healthy dietary choices and physical activity in 10–12 year olds in an RCT using a commercial-quality computer game specifically designed to incorporate social cognitive, self-determination, and persuasion theories [36]. Their intervention was successful at improving dietary choices. Silk *et al*. examined the effectiveness of print, a website, and a computer game to impact nutrition education in a group of female adults with the website and game modalities faring better than print on attention, and the website ranking highest among the three modalities for attention, understanding of content, learning, and intent to use for additional information [37]. The authors note that the results may be indicative of the preferences of the audience and the appropriateness of games in the domain of the study. This is an important point, as the benefits of games seen in some areas of study with some populations may not translate to all intervention domains or audiences. Additionally, the design and quality of the game can be instrumental to a successful intervention [38–40].

In the domain of energy there have been a number of games targeted at energy efficiency behavior. Orland *et al*. implemented an RCT targeted at reducing energy consumption in the workplace [41]. Sensors were used to measure and provide feedback through a game interface with short-term reductions in energy consumption, but these energy savings did not persist in the eight weeks following game play. Reeves *et al*. developed a commercial quality game that proved effective in reducing energy use by 2% amongst the college student participants in their RCT study, but the savings did not persistent in the 30-day follow-up [40]. Gustafsson *et al*. found both increased energy efficiency behavior and increased attitude toward energy efficiency in their RCT study with 12–14 year olds, but the significance of difference between game players and the control group diminished shortly after the game [42]. While the effectiveness of games in reducing energy consumption is encouraging, the lack of persistence presents a challenge to interventions targeting ongoing behavior change. This suggests that such interventions may prove more effective with one time behaviors, such as energy efficiency upgrades or solar adoption, that do not need to be repeatedly sustained beyond the adoption event.

Given the variability of results seen across so many studies, we sought to mitigate potential ambiguities by: 1) employing a relatively simple game design (compared to designs such as multi-level, simulation, virtual world), with 2) a singular emphasis on information delivery, 3) in comparison to a standard mode of communication, 4) using an RCT design and repeatability of results. As our goal is to examine the impact on behavior, which will be measured by self-reported performance of that behavior, we have selected a specific and discrete behavior with a singular path to action (i.e., calling a solar installer to receive a quote and eventually have solar installed). Our participants are adults living in single-family homes, who are, thus, a suitable population for the behavior under study, especially in comparison to student populations (not usually in a position to adopt solar) and employees (not acting in the context of personal decision making) that typically make up serious game and gamification study participants.

## Methods

### Survey Instrument

Both of the experiments in this study use the same survey instrument (with the exception of one question discussed below) based on the TPB framework to assess the impact of attitudes, normative beliefs, and perceived behavioral control on intentions and behavior related to residential solar adoption and how those constructs change in relation to passive and interactive information interventions. This survey instrument is an expanded version of the survey instrument used in our previous research [43], which was developed using the guidelines for a TPB questionnaire [21,25,44]. Based on the prior results, we conducted a salient beliefs survey and further refined the survey instrument, keeping the original questions and adding more. By using the same questions in both experiments discussed in this paper, we are able to make direct comparisons between the two experiments. Additionally, the survey was administered during the fall (September and October) of the same year for both experiments, thus reducing sensitivity to high summer and winter bills.

The survey uses a 7-point bipolar Likert scale with “Agree” written by 7 and “Disagree” written beside 1, unless otherwise noted. The survey addresses measures of attitudes, norms, PBC, intentions, and behavior with respect to residential solar energy, as well as demographic data. Each TPB construct is measured using multiple questions selected via exploratory factor analysis and represented by an index variable calculated as the mean of responses to those questions. The full list of questions used for each variable is available in S1 File.

Attitude toward solar was measured as a composite of the overall appeal of solar, expected cost savings, expected effect on home value, visual appeal (positive and negative framing), and environmental impact of solar. The subjective norms index variable was formed from two questions asking if those people who are important to the participant would support installing solar and would approve of installing solar. PBC includes the ease of installing solar, the perceived affordability, knowing what steps to take, and having the time to have the system installed. Intention toward solar was measured by asking the likelihood of calling a solar installer to request a quote. Behavior was measured through actually calling a contractor or solar installer to request a quote. We did not measure solar adoption as a behavior, since participants would not have had enough time to have solar installed (typically a few months) given the short duration of the experiment (approximately two weeks).

The survey used for the passive information campaign also included a general measure of familiarity with solar energy; this was the only difference between the survey instruments used for the experiments. Additional questions asked participants about awareness of solar incentives, energy use and efficiency upgrades, and both intention and behavior toward energy audits. The content included in both the passive and interactive information campaigns includes energy efficiency information as a way to provide useful information to a broader range of participants, potentially reducing selection bias toward those exclusively interested in solar. Survey questions about energy use and audits serve the same purpose as the energy content. These studies are IRB exempt under the Sandia National Labs Human Studies Board (SNL1520, see S1 Memo), and the participant consent statement is available in S1 File.

### Content

The content for both experiments included solar energy, as well as energy efficiency, in order to appeal to a broader audience and reduce selection bias amongst solar enthusiasts. The solar energy content included basic solar literacy content (technology basics, cost, leasing options, local and federal incentives), while the energy efficiency content of the game was based on Gardner and Stern’s “short list” of the most effective energy conservation tools (complete content available in S2 File)[45]. Participants in both experiments received the same information, with format details discussed in the respective experimental design sections below. The information focused on actionable information easily presented through the trivia-style game format. Since subjective norms by their nature of being subjective are harder to convey in factual trivia-style questions, the information favors content related to attitudes and PBC. Due to the limited content on norms, the emphasis of our analysis is on attitudes and PBC.

## Passive Information: Amazon Mechanical Turk RCT

### Experimental Design

**Fig 1:**
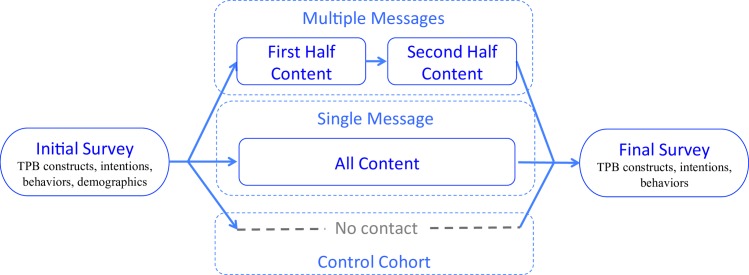
Experimental design of passive information experiment. Multiple and single message groups receive the same content, except that the former receives it in two smaller pieces. The “first half content” and “second half content” together are exactly the same as “all content” received by the single message cohort.

As noted above, the experimental design includes a pre- and post-survey to capture attitudes, subjective norms, PBC, intentions, and behavior before and after the treatment with a 17 day gap between surveys for the AMT passive information experiment. The time between the two surveys was designed to approximate the timing of Energy Games (the interactive information experiment described below). The AMT passive information experiment has three cohorts as shown in Fig 1 below: control (survey only, no information), single message (single), and multiple (but unrepeated) messages (multi) of the same information. The control group received no communication between the surveys. The two treatment groups received the same information on solar energy and energy efficiency as provided in the Energy Games experiment, which is discussed in more detail below. However, the single group received all the information at a single point mid-way (day 8) between the two surveys, whereas the multi group received the same content as the single group, but broken into two sections delivered at approximately equal intervals (day 4 and day 8). Each of the two sections was delivered only once to the multi group, i.e., no information was repeated.

The two treatment cohorts received the content in the form of information surveys (see S2 File) with the content followed by two Likert scale questions: “The information provided here was useful to me,” and “The information provided here was new to me.” These questions were intended only to confirm that participants clicked through the information. The information survey concluded with a page of links to more information, corresponding to links provided in Energy Games. Only responses from participants that completed all HITs for their cohort were retained in the final data. Respondents were compensated $1 per pre- or post-survey, $1 for the single message information survey, and $0.50 for each of the multiple message information surveys. Thus both treatment groups received the same total compensation for reviewing the same content.

This experiment was conducted as an RCT through AMT, where respondents were first screened for location in the U.S. and owning a single family home. After pre-screening, 699 qualified respondents were invited to complete the pre-survey. Based on prior experience, we planned for retention of approximately 75% at each contact point [46]. Thus, the total of 524 responses was randomly assigned to one of the three groups, with 25% assigned to the control, 25% to the single message condition, and 50% to the multi message condition. The multi group had a larger initial allocation to allow for greater attrition and fatigue due to the additional contacts for the multiple content messages. After the post survey, the three cohorts had 117 (90% retention), 68 (52%), and 109 (42%) respondents for the control, single, and multi groups, respectively, that completed all the HITs for their respective cohort. The higher retention rate for the control group may be due to the higher HIT payment for the pre- and post-surveys compared to the typical AMT task. Mason & Watts (2009) demonstrated that higher payments increased the completion of HITs, but did not influence the quality of work [47]. We note that the main purpose of the incentives was to encourage retention among the respondents and to ensure a robust control. Because this study does not involve an economic experiment, in the sense that the provision of monetary incentives are not associated with expected changes in the studied behavior (such as changes in TPB constructs), the provision of monetary incentives does not interact with changes in the variables of interest in the study.

### Results

#### Demographic analysis

The three cohorts show no significant differences along demographic variables, which include age, gender, household income, educational attainment, home area, and home value. The mean age of participants is 38 years (sd = 12 years), with 50% female respondents. The modal and median incomes are between $50k-$75k. The modal educational attainment is a Bachelor’s degree (42%), with 35% having lower educational attainment and 22% higher. The average home is 1820 sq ft (sd = 960 sq ft) and costs $215k (sd = $180k).

#### Survey analysis

Table 1 shows the mean response for Likert scale items and proportion responding “yes” for yes/no questions on both the pre- and post-survey, followed by the change in the means (or proportion) between the two surveys by cohort. The final three columns show the difference in the change in the mean, pairwise between cohorts. The significance of the change was determined using two-way repeated measures ANOVA with cohort as the between subjects variables and time as the within subjects variable, controlling for age, gender, household income, educational attainment, home area, and home value. Participants in the single cohort spent a mean time of 5.1 minutes on the information survey, while the multi cohort spent a mean total time of 6.7 minutes (3.3 minutes on the first and 3.4 minutes on the second information survey). This time does not include time spent taking the pre- and post-surveys. The difference in time spent by the multi cohort represents a statistically significant increase over the single cohort (t(158) = 2.40, p = 0.02).

**Table 1:** Passive information survey results. Pre- and post-survey means by cohort (standard deviation below), within cohort change in mean survey score (standard deviation below), and pairwise difference in the within cohort change between cohorts. For binomial variables the proportion responding ‘yes’ or the change in proportion of ‘yes’ responses. Index variables show Cronbach’s alpha in ().

	pre-survey			post-survey			within cohort change			between cohort post-pre					
	control.0	single.0	multi.0	control.1	single.1	multi.1	Δcontrol	Δsingle	Δmulti	single-control		multi-control		multi-single	
**Familiar**	4.2	4.04	4.06	4.23	4.49	4.52	0.03	0.44	0.47	**0.41**	**	**0.44**	*	0.03	
Familiar.sd	1.4	1.33	1.52	1.35	1.31	1.37	1.31	1.38	1.39						
**SA** (α(.78)	5.4	5.18	5.29	5.48	5.18	5.25	0.08	0	-0.04	-0.08		-0.12		-0.04	
SA.sd	0.88	0.83	0.93	0.78	0.88	0.98	0.57	0.65	0.55						
**SSN** (α(.9)	5.52	5.26	5.7	5.55	5.28	5.74	0.03	0.02	0.04	-0.01		0.01		0.02	
SSN.sd	1.2	1.39	1.17	1.3	1.24	1.13	0.97	1.01	1.08						
**SPBC**(αP.75)	3.69	3.71	3.54	3.78	3.87	3.95	0.09	0.16	0.42	0.07		**0.33**	**	**0.26**	*
SPBC.sd	1.26	1.26	1.17	1.41	1.12	1.2	0.96	0.97	1.02						
**SIquote**	3.02	2.89	2.85	2.92	3.22	3.26	-0.1	0.33	0.4	**0.43**	.	**0.5**	*	0.07	
SI.sd	1.66	1.59	1.77	1.62	1.63	1.73	1.39	1.32	1.37						
	**proportion responding yes**							**change within**			**change between**						
**SBquote**^a^	0.13	0.16	0.08	0.13	0.21	0.14	0	0.05	0.06	0.05		0.06		0.01	

note:.p < .1

*p < .05

**p < .01

***p < .001.

^a^ Dichotomous variables analyzed using McNemar’s test for within cohort change and generalized linear mixed model for between cohort change.

Both the single (F(1, 236) = 6.95, p = 0.009) and multi (F(1, 236) = 4.1, p = 0.04) cohorts show a significant change in familiarity with solar compared to the control, with a mean increase of 0.41 and 0.44, respectively, over the change in familiarity for the control group (0.03). The effect size is 0.32 for the multi cohort compared to the control and 0.3 for the single cohort compared to the control, calculated using Cohen’s d [48]. This indicates that both information treatments are equally effective at increasing a general, non-specific sense of familiarity with the topic, with a small effect size.

For the more specific metrics relating to the TPB constructs, PBC toward solar among the three cohorts shows a significant increase (F(2, 236) = 4.8, p = 0.009), while attitude and subjective norms toward solar indicate no significant changes (F(2, 236) = 0.55, p = 0.58 and F(2, 236) = 0.30, p = 0.74, respectively). The pairwise post hoc analysis confirms a significant increase in PBC for the multi cohort compared to both the control and the single cohort (F(1, 236) = 8.26, p = 0.004 and F(1, 236) = 5.56, p = 0.02, respectively) with an increase in mean of 0.33 and 0.26 and an effect size of 0.33 and 0.26, respectively. The single cohort shows no significant increase in PBC compared to the control group (F(1, 236) = 0.02, p = 0.9). These results suggest that more frequent, smaller amounts of information may be more effective for positively impacting solar PBC. This is noteworthy, since of the three TPB constructs PBC is the most important one to impact, based on prior TPB models that indicate that PBC has the greatest influence on intention and behavior for solar energy [43].

Given the relatively short length of the study (two weeks), measuring solar adoption was infeasible since the process of installing solar takes longer than two weeks. Instead, we measured the behavior of calling a solar installer for a quote (SBquote), an essential step in the final adoption decision, and the intention of that behavior as likelihood of calling an installer for a quote (SIquote). Solar intentions change significantly among cohorts (F(2, 200) = 3.21, p = 0.04). The multi cohort shows a significant increase in intentions toward solar, with a mean increase over the control group of 0.3 (F(1, 200) = 5.29, p = 0.02) and effect size d = 0.36. The single cohort shows a marginally significant increase over the control group with a mean increase of 0.28 (F(1, 200) = 3.53, p = 0.06) and effect size d = 0.32. There is no significant difference between the multi and single cohorts (F(1, 200) = 0.02, p = 0.9) regarding intentions. Both the single and multi cohorts show an increase in solar quote behavior relative to the control, with increases of 5% and 6%, respectively; however, these increases are not statistically significant (see Table 1). Furthermore, the single and multi cohorts did not differ from each other in solar quote behavior. The similarity of the results between the two treatment cohorts for both solar intentions and behavior indicates that changes in intentions were not related to changes in PBC in this experiment. While we normally would have expected a higher increase in intentions and behavior for the multi cohort, due to the increase in PBC, in this case the initial low value of PBC (3.54 out of 7) only increases to 3.95, still below the neutral mark (4.0 out of 7). This may not be high enough to activate changes in intention or behavior. Additionally, TPB models of SIquote using the pre-survey indicate that the behavioral antecedents account for approximately 28% of variance, which is further supported by models of the same variable in Rai & Beck (2015) showing 24% of variance explained (n = 417)[43]. Thus the effect responsible for the change in intentions and behavior in response to the passive information is likely explained by the remaining 70% of variance. For instance, information salience, or increased resonance of solar information, resulting from the information treatments could be driving increases in intentions toward the behavior.

## Interactive Information: Energy Games within subjects design

### Experimental Design

This experiment was based on our previous Energy Games RCT [16]; however, this experiment includes a within subjects repeated measures design only, since the method of recruitment did not permit the formation of a robust control group. To address this weakness to some extent in discussing the results below, we compare with the results in Rai and Beck (2016), which did have a control group and uses the same content and nearly the same pre/post survey design.

**Fig 2:**
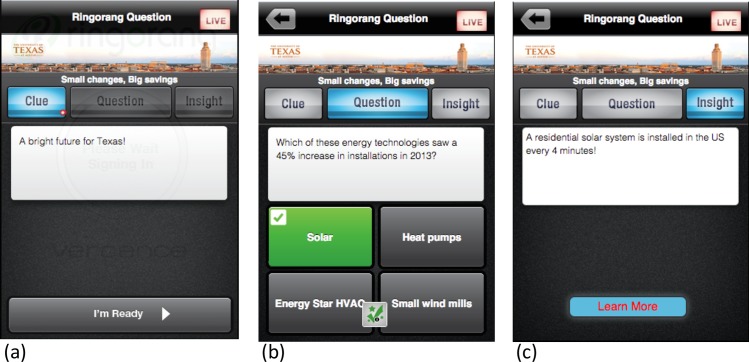
Ringorang^®^ screen shots. Screen shots of the Ringorang^**®**^ question format a) clue, b) question, and c) insight.

Energy Games is an interactive trivia-style game for mobile devices and PCs based on the Ringorang^®^ platform, which supports customizable content. As shown in Fig 2, the question is presented in an interactive sequence that includes a clue, followed by a question and multiple-choice answer options. While the results are computed in real-time, players are presented an insight that elaborates the subject matter with an optional link (“Learn More”) for additional information. The app then reveals the correct and incorrect answers.

All players in Energy Games receive questions simultaneously, one question at a time, spaced throughout the day. When a question is delivered, the app alerts players–everyone at once–and the player can then choose to play the question or ignore the prompt (with the option to play later). Players do not have to play in real-time; they also have the option to play questions in make-up mode, at their convenience, through the end of the week. Make-up mode presents the clue, question, and insight exactly as in the real-time mode.

We recruited participants in partnership with the North Texas Renewable Energy Group, a member organization of the Texas Solar Energy Society, in conjunction with their outreach efforts for the Dallas-Ft. Worth annual solar tour. Messaging during recruitment focused on energy efficiency and solar energy, as a means of reducing selection bias towards those specifically interested in solar energy, as well as on prizes, which included $10 instant win prizes (no more than one per player per week), weekly leaderboard prizes ($50 for first, $35 for second, $25 for third, $15 for fourth, and $10 for fifth), and a grand prize drawing of a $500 Ikea gift card. The main purpose of the incentives was to encourage respondents to sign up for and play Energy Games. As in the passive information experiment, because Energy Games is not an economic experiment, the provision of monetary incentives does not interact with changes in the variables of interest in the study, i.e. the TPB constructs.

Interested participants were directed to the pre-survey described in the Methods Section and given instructions for registering and downloading the game. A total of 135 participants completed the pre-survey, 83 registered for the game, 59 played the game, and 53 completed the final survey, leading to an overall 39% retention rate. Of the final 53 participants, eight were removed from the analysis because they had already installed solar, thus had already acted on the behavior the experiment was designed to influence. This leaves a final sample size of 45 participants in the Energy Games experiment. Following the pre-survey, participants played Energy Games for two weeks between September 14–27, 2015, leaving one week to complete the post-survey before the tour on October 3, 2015. The survey closed prior to the solar tour date in order to ensure that the game, rather than the tour, was the primary source of solar information.

For Energy Games the content was organized into two themes (low effort and high effort), each lasting one week for a total of 15 questions per week (five questions per day, three days a week) roughly split on solar energy and energy efficiency each week. Overall, the Energy Games experiment lasted for two weeks and entailed 30 questions. These questions covered the same content as the information surveys provided to the passive information study participants.

### Results

#### Demographic analysis

Demographic variables include age, gender, household income, educational attainment, home area, and home value. The average mean age of participants is 42 years (sd = 10 years) with 33% female. The modal income is between $75k-$100k. Educational attainment is a Bachelor’s degree for 75% of respondents, with the remaining 25% having a post-graduate degree. The average home is 2600 sq ft (sd = 860 sq ft) and costs $220k (sd = $125k), with a Pearson correlation of r = 0.75.

#### Survey analysis

The mean response and standard deviation for Likert scale items and percentage responding yes/no for binomial questions on both the pre- and post-survey, followed by the change in the means (or percentage) between the two surveys are shown in Table 2. The significance of the change was determined using a paired t-test and effect size (Cohen’s d). For binomial response questions, McNemar’s test was used to determine significance of the within subjects change between the pre- and post-surveys. Energy Games participants engaged for a mean total time of 18.6 minutes over the course of two weeks (8.4 minutes in week one and 10.2 minutes in week two), not including the time spent taking the pre- and post-surveys.

**Table 2:** Energy Games survey results. Pre- and post-survey means and standard deviations, change between surveys, and Cohen’s d measure of effect size. For binomial variables, the proportion responding no/yes and change between proportions responding yes are shown. Index variables show Cronbach’s alpha in ().

	pre-survey		post-survey		change			effect size
	mean.0	sd.0	mean.1	sd.1	Δmean		sd	d
**SA** (a = .75)	5.23	0.72	5.49	0.85	0.26	*	0.83	0.32
**SSN** (a = .83)	5.42	1.01	5.43	1.16	0.01		1.11	
**SPBC** (a = .89)	3.9	1.09	4.56	0.99	0.66	***	1	0.66
**SIquote**	4.11	1.56	4.72	1.65	0.61	***	0.96	0.63
	**No**	**Yes**	**No**	**Yes**	**Δproportion **			** **
**SBquote**	0.87	0.13	0.8	0.2	0.07			

note:.p < .1

*p < .05

**p < .01

***p < .001.

Both solar attitude (t(44) = 2.12, p = 0.04) and PBC toward solar (t(44) = 4.43, p = .0001) show a significant increase, while subjective norms toward solar indicate no significant change (t(44) = 0.07, p = 0.9). Solar attitude showed a mean increase of 0.26 points, with a small to medium effect size, d = 0.32. PBC saw a mean increase of 0.66 with a slightly greater than medium effect size d = 0.66. These results are in agreement with the previous Energy Games RCT results that had a medium to large effect size (d = 0.71) for increase in PBC [16]. As discussed above, PBC is the most important antecedent to impact as prior TPB models indicate that PBC has the greatest influence on intention and behavior for solar energy [43]. Thus, these results suggest that for PBC the gamified information was more effective than the passive information cohorts, which experienced only half the change in PBC.

Energy Games participants also show a significant increase in intentions, i.e., likelihood of requesting a quote (SIquote), toward solar with a mean increase of 0.61 (t(35) = 3.8, p = .0006) and an effect size of 0.63. This result is also in agreement with the prior Energy Games RCT, where solar intentions increased by 0.68 compared to the change in the control with an effect size of 0.49. Additionally, this effect size is nearly twice that for both information treatment cohorts in the passive information experiment (0.32–0.36). For solar behavior, SBquote, the percentage change is similar to the passive information study; however, the sample size for Energy Games is less than half that for the multi cohort. Thus, the absolute change is not large enough to register a significant change in behavior. Given that Energy Games was promoted with a community solar tour, it is also possible that participants were waiting until after the tour to move forward on calling an installer.

While the different designs of the passive and interactive information experiments can make direct comparison difficult, the consistency of results for Energy Games, here and in our prior RCT, makes this comparison more reliable. The prior Energy Games RCT used the same content (though specific program information reflected the local utility or relevant information source) and pre/post survey design, showed an increase in PBC and intention toward solar for the game cohort compared to the control, and resulted in similar effect sizes to the study reported here, thus supporting the results of the within subjects design.

## Conclusion

We investigated the impact of both passive and interactive approaches to information delivery on the TPB antecedents (attitudes, subjective norms, PBC) of intentions and behavior, as well as their effect on intentions and behavior directly, with a focus on solar energy adoption. That PBC influences behavior both directly and indirectly, through intentions, indicates that impacting PBC has the greatest potential for a durable influence on intentions and, eventually, behavior. Thus the medium to large effect size for increasing PBC seen in the interactive information experiment is particularly promising. The small effect size for PBC in the multi message cohort, particularly compared to no change for the single message cohort, suggests that smaller, more frequent interaction may prove to be a more effective means of communicating complex, multi-faceted information.

The results of our study indicate that both the single and multi message treatments increased familiarity with solar energy information, intentions toward solar, and a small (~5% over two weeks), but statistically insignificant, increase in the solar behavior of requesting a quote. However, only the multi message condition significantly impacted solar PBC compared to the control. This effect across these key behavioral attributes (in TPB) indicates greater potential for delivering smaller amounts of information more frequently. One limitation of our study is that we did not “test” participants for knowledge gain or accuracy of recall, as our primary interest was in how the information delivery mode impacted perceptions and intentions, rather than exploring which mode is most effective for learning. A difference in learning outcomes between the modes, if it exists, could explain why familiarity increased for both groups, while only the multi cohort showed a significant increase in PBC, which is a more specific metric.

The greater impact of the multi message condition over the single message condition could be related to a number of possible effects. Both groups received the exact same content in total and received that content only once (no repetition), but the single cohort spent less time engaging with the content compared to the multi cohort. Thus more, smaller batches of information may result in greater overall exposure to the content. This seems likely since the multi cohort spent less time per information survey, but more time cumulatively. Additionally, the multiple messages may bring more salience to the topic due to being prompted twice to think about solar energy instead of only once for the single cohort. To the extent that these aspects of multiple messages improves the efficacy of information, Energy Games would further enhance the effect since participants received the information with more frequency and shorter duration, and spent nearly three times as long engaging with the same content.

Energy Games participants exhibited a statistically significant increase in attitude, PBC, and intentions toward solar. These results were in agreement with our previous RCT study of Energy Games [16]. Moreover, the effect size of the change in PBC and intentions was nearly twice that for the multi cohort, thus the interactive information showed increased impact over the passive information study, which may be related to the even smaller and higher frequency delivery of the content.

Overall, results from the two experiments in this study support the conclusion that the mode of information delivery is a significant factor in the time engaged, content consumed, and the subsequent impact that information has on the antecedents of behavior. This finding has practical implications. For example, currently, passive information is the predominant mode of communication for energy utilities. While there may be passive delivery modes that could replicate the frequency of Energy Games, the motivational affordances provided by serious games provides a means of engaging and retaining participants, a critical component as information alone is not sufficient [1,49,50]. We note that the intention and behavior measured here are those of likelihood to call for a quote and calling for a quote, respectively, rather than solar adoption. Given the short duration of the experiment (approximately two weeks), installing solar is an unlikely outcome within this duration, thus further work will be required to determine the long-term impact and durability on behavior change, such as solar adoption. However, our results, which are unique in using a diverse adult population (as opposed to captive audience of employees or students), are encouraging for improving the design of behavioral interventions that can increase information salience and have impact on a wider scale.

## Supporting Information

S1 File This document contains the full survey content used in the experiments. (PDF)

S2 File This document includes the content provided for the passive information study and Energy Games. (PDF)

S1 Data This document includes a csv of the survey data used to generate results. (CSV)

S1 Memo This document is a memo stating exemption from the Sandia Labs Human Studies Board. (PDF)
