# Supplementary material for: Small Is Big: Interactive Trumps Passive Information in Breaking Information Barriers and Impacting Behavioral Antecedents
Source: PLoS One. 2017 Jan 18;12(1):e0169326. doi: 10.1371/journal.pone.0169326 (PMC5242502; doi:10.1371/journal.pone.0169326)
Supplement: S1 File — (PDF) [file pone.0169326.s001.pdf]

S1 Supporting Information: Surveys

Small is Big: Interactive trumps passive information in breaking information barriers and impacting behavioral antecedents

Ariane L. Beck[1*], Kiran Lakkaraju[2], and Varun Rai[1,3]

Survey Questions

Participant Consent:

This survey is part of a study on communicating energy conservation and solar energy information.

Thank you for taking our survey on solar energy as part of a research study from Sandia National Labs and The University of Texas at Austin to help us learn the best ways to communicate solar energy and energy efficiency information to electric customers. This survey should only take 5-10 minutes.

Your responses will be kept completely confidential. Responses and contact information will not be shared with any third parties.

Participation is voluntary. By beginning the survey, you acknowledge that you have read this information and agree to participate in this research, with the knowledge that you are free to withdraw your participation at any time. If you do not want to continue, you can simply leave this website.

Solar energy measures:

Familiarity. How familiar are you with residential solar energy installations?

Attitudes. 1) Having a solar system installed on my home would overall be... (Unappealing/Appealing); 2) I think a solar energy installation would save me money; 3) I think a solar energy installation would increase the value of my home; 4) I think a rooftop solar installation would increase the visual appeal of my home. 5) I think rooftop solar installations are ugly. 6) Solar energy is good for the environment.

Subjective Norm. 1) If I install a solar system on my roof, people who are important to me would approve. 2) Most people who are important to me would support my installing solar.

[1] LBJ School of Public Affairs, The University of Texas at Austin, Austin, TX 78712
[2] Sandia National Labs
[3] Mechanical Engineering Department, The University of Texas at Austin, Austin, TX 78712
[*] e-mail: abeck@utexas.edu

Perceived Behavioral Control. 1) Having a solar system installed on my roof would be... (Difficult/Easy) 2) A solar system is affordable for my household. 3) If I choose to install solar, I'm confident I know what steps to take. 4) If I choose to install solar, I have the necessary time to do so.

Intention. How likely is it that you will request a quote for a solar installation some time in the near future (within the next few months)?

Behavior. 1) Have you ever requested a quote for a solar installation? (Yes/No, pre); Since taking the initial survey, have you requested a quote for a solar installation? (Yes/No, post)

Incentive Awareness. Are you aware of any incentives (federal, state, or local) to install solar? (pre); Since taking the initial survey, have you become aware of any incentives (federal, state, or local) to install solar? (post)

Energy conservation measures:

Intention. 1) How likely are you to make changes to how you use energy to save energy at home (thermostat setting, unplugging electronics, etc.) over the next few months? 2) How likely are you to make energy upgrades to your home (energy efficient appliances, weatherization, insulation, etc.) over the next few months? 3) How likely is it that you will have an energy audit on your home in the near future (within the next few months)?

Behavior. 1) Have you made any changes to how you use energy to save energy at home (thermostat setting, unplugging electronics, etc.) in the recent past (the last few months)? (Yes/No, pre); Since taking the initial survey, have you made any changes to how you use energy to save energy at home (thermostat setting, unplugging electronics, etc.)? (Yes/No, post); 2) Have you made any energy upgrades to your home (energy efficient appliances, weatherization, insulation, etc.) in the recent past (the last few months)? (Yes/No, pre); Since taking the initial survey, have you made any energy upgrades to your home (energy efficient appliances, weatherization, insulation, etc.)? (Yes/No, post) 3) Have you had an energy audit on your home? (Yes/No, pre); Since taking the initial survey, have you had an energy audit on your home? (Yes/No, post)

Environmental concern:

In general, I am concerned about environmental issues.
People need to change their lifestyles to protect the environment.
