# Supplementary material for: Small Is Big: Interactive Trumps Passive Information in Breaking Information Barriers and Impacting Behavioral Antecedents
Source: PLoS One. 2017 Jan 18;12(1):e0169326. doi: 10.1371/journal.pone.0169326 (PMC5242502; doi:10.1371/journal.pone.0169326)
Supplement: S2 File — (PDF) [file pone.0169326.s002.pdf]

S2 Supporting Information: Content

Small is Big: Interactive trumps passive information in breaking information barriers and impacting behavioral antecedents

Ariane L. Beck[1*], Kiran Lakkaraju[2], and Varun Rai[1,3]

1.0 Passive Information Content

1.1 Single message

Solar Energy
Please read the following key points on solar energy and answer the following questions.

- A solar PV system is installed in the US every 2.5 minutes! The number of Solar PV installations increased over 50% this past year.
- Many solar leases require no down payment, include free maintenance, and monthly lease payments will be more than covered by reduced electric bills with cash to spare.
- Solar can be installed on most roof types or ground mounted and can face any direction, except north. Ask a solar professional to assess your home's potential and recommend the best design for your home.
- Solar panel prices have dropped almost 50% in the last 5 years, but prices are expected to level off in the near future, while a lot of incentives and rebates are being phased out. That makes this a great time to invest in solar.
- In many areas, a solar installation can pay for itself in less than 10 years, while producing electricity for over 30 years, making it quicker and easier than ever to get a good return on your investment.
- Live a drought prone area? Neither solar PV nor wind power use water to produce energy, which makes renewable energy a great option for cities prone to drought or where water is scarce.
- The Investment Tax Credit provides an incentive of 30% off the cost of a solar PV system. Many local utilities offer additional rebates and incentives. Check the Local Incentives quick link on the last page for more info.
- After 25 years, solar panel output will decline slightly, but the panels will still produce power. Most solar panels come with 20-30 year warranties to guarantee it.
- On a cloudy day solar panels produce less electricity, but they still produce. Don't worry, if you need more energy, the grid has you covered.
- Many electricity providers will pay you for the extra energy you produce, but you may have to do a little research as policies change by state and utility. Check with your local utility for more information.
- While producing energy for your home, solar panels can also reduce your cooling bill by absorbing the sun's heat, providing shade and keeping your house cool.

[1] LBJ School of Public Affairs, The University of Texas at Austin, Austin, TX 78712
[2] Sandia National Labs
[3] Mechanical Engineering Department, The University of Texas at Austin, Austin, TX 78712
[*] e-mail: abeck@utexas.edu

- You can't test-drive a solar installation, but talking to the neighbors can provide a lot of valuable experience and insight that can help you decide. Don't be shy, our research shows solar owners are very happy with their decision and love to share info on solar.

Rate from 1 to 7 how much you disagree or agree with each of the following statements.
The information provided here was useful to me.
The information provided here was new to me.

Energy Conservation
Please read the following key points on energy conservation and answer the following questions.

You can cut your electric bill up to 25% with simple energy efficiency tips. Big energy users are a great place to start saving, and heating and cooling are the biggest energy users for most homes.

Programmable Thermostats

- Programmable thermostats make it easy to save and can cost as little as $20 at your local hardware store.
- Setting the AC thermostat to 78°F in the summer and 68°F in the winter. You can save 3% off your electric bill for each degree you increase/decrease (for cooling/heating) your thermostat setting. Check the Energy Star recommended settings provided in the "Keeping Cool" Quick Link on the next page of this survey.
- Like coming home to a cool house? A programmable thermostat can start cooling your house before you get home.
- Remember… To cool down your house to 78F, setting the thermostat to 70F does not cool it faster than setting it to 78F. Program it to start cooling about a half hour before you usually get home.

An **AC Tune-up** can save up to 30% off the cost of cooling your home. If it's time to replace your HVAC, look for the Energy Star – it could save you $200 per year. Oversized air conditioners can leave your home humid. Size your AC right to keep humidity, costs, and energy use lower!

Ceiling Fans can save money on cooling, by making the room feel about 5 degrees cooler. The trick with ceiling fans is to remember to turn them off when you leave the room.

Weatherize your home! Adding insulation, sealing ductwork, and weather stripping doors and windows are inexpensive ways to save energy for the long term. You can reduce cooling costs 10%-25% by weatherizing your home. Weatherizing is an easy and inexpensive DIY project that can cost as little as $5.

Energy Star appliances save on energy and water!
Use that **Dishwasher**! ENERGY STAR dishwashers annually save you 5,000 gallons of water, $40 on your energy bill, and 230 hours (that you get to spend NOT washing dishes).

Hot Water Heaters set at 120°F will save energy without sacrificing comfort. You'll still have plenty of hot water. Check your water heater today and turn it down if it's above 120F. Some water heaters mark 120F with an arrow for the optimal setting.

Clothes Washer water temp set to warm or cold can save half the cost of washing a load of laundry – 90% of energy used for washing clothes is used heating the water. An ENERGY STAR washer saves about 20% of the energy and 35% of the water over other models.

LED Lighting can save $60/year by replacing 5 incandescent bulbs with LED bulbs. LEDs are more efficient and last 5 times longer than CFLs. You don't have to replace them all at once, start with the ones you use the most.

Refrigerators run more efficiently if kept full, set to 38F, and the seals are tight. Refrigerators and freezers are more efficient when full. Use water bottles to fill up extra space.

Stop Vampire Power! The average American household spends about $100 every year powering electronics when they're turned 'off'. Unplug power blocks when not in use. About 10% of home electricity costs are wasted on vampire power - that's all the electricity used by all of Italy in one year.

Rate from 1 to 7 how much you disagree or agree with each of the following statements.
The information provided here was useful to me.
The information provided here was new to me.

Quick Links were provided at the end of the survey with the same links provided in the "learn more" items in Energy Games.

1.2 Multiple messages

1.2.1 Week 1

Solar Energy
Please read the following key points on solar energy and answer the following questions.

- A solar PV system is installed in the US every 2.5 minutes! The number of Solar PV installations increased over 50% this past year.
- Many solar leases require no down payment, include free maintenance, and monthly lease payments will be more than covered by reduced electric bills with cash to spare.
- Solar can be installed on most roof types or ground mounted and can face any direction, except north. Ask a solar professional to assess your home's potential and recommend the best design for your home.
- Solar panel prices have dropped almost 50% in the last 5 years, but prices are expected to level off in the near future, while a lot of incentives and rebates are being phased out. That makes this a great time to invest in solar.
- In many areas, a solar installation can pay for itself in less than 10 years, while producing electricity for over 30 years, making it quicker and easier than ever to get a good return on your investment.
- Live a drought prone area? Neither solar PV nor wind power use water to produce energy, which makes renewable energy a great option for cities prone to drought or where water is scarce.

Rate from 1 to 7 how much you disagree or agree with each of the following statements.
The information provided here was useful to me.
The information provided here was new to me.

Energy Conservation
Please read the following key points on energy conservation and answer the following questions.

You can cut your electric bill up to 25% with simple energy efficiency tips. Big energy users are a great place to start saving, and heating and cooling are the biggest energy users for most homes.

Programmable Thermostats

- Programmable thermostats make it easy to save and can cost as little as $20 at your local hardware store.
- Setting the AC thermostat to 78°F in the summer and 68°F in the winter. You can save 3% off your electric bill for each degree you increase/decrease (for cooling/heating) your thermostat setting. Check the Energy Star recommended settings provided in the "Keeping Cool" Quick Link on the next page of this survey.
- Like coming home to a cool house? A programmable thermostat can start cooling your house before you get home.
- Remember… To cool down your house to 78F, setting the thermostat to 70F does not cool it faster than setting it to 78F. Program it to start cooling about a half hour before you usually get home.

An **AC Tune-up** can save up to 30% off the cost of cooling your home. If it's time to replace your HVAC, look for the Energy Star – it could save you $200 per year. Oversized air conditioners can leave your home humid. Size your AC right to keep humidity, costs, and energy use lower!

Ceiling Fans can save money on cooling, by making the room feel about 5 degrees cooler. The trick with ceiling fans is to remember to turn them off when you leave the room.

Weatherize your home! Adding insulation, sealing ductwork, and weather stripping doors and windows are inexpensive ways to save energy for the long term. You can reduce cooling costs 10%-25% by weatherizing your home. Weatherizing is an easy and inexpensive DIY project that can cost as little as $5.

Rate from 1 to 7 how much you disagree or agree with each of the following statements.

The information provided here was useful to me.
The information provided here was new to me.

Quick Links were provided at the end of the survey with the same links provided in the "learn more" items in Energy Games.

1.2.2 Week 2

Solar Energy
Please read the following key points on solar energy and answer the following questions.

- The Investment Tax Credit provides an incentive of 30% off the cost of a solar PV system. Many local utilities offer additional rebates and incentives. Check the Local Incentives quick link on the last page for more info.
- After 25 years, solar panel output will decline slightly, but the panels will still produce power. Most solar panels come with 20-30 year warranties to guarantee it.

- On a cloudy day solar panels produce less electricity, but they still produce. Don't worry, if you need more energy, the grid has you covered.
- Many electricity providers will pay you for the extra energy you produce, but you may have to do a little research as policies change by state and utility. Check with your local utility for more information.
- While producing energy for your home, solar panels can also reduce your cooling bill by absorbing the sun's heat, providing shade and keeping your house cool.
- You can't test-drive a solar installation, but talking to the neighbors can provide a lot of valuable experience and insight that can help you decide. Don't be shy, our research shows solar owners are very happy with their decision and love to share info on solar.

Rate from 1 to 7 how much you disagree or agree with each of the following statements.
The information provided here was useful to me.
The information provided here was new to me.

Energy Conservation
Please read the following key points on energy conservation and answer the following questions.

Energy Star appliances save on energy and water!

Use that **Dishwasher**! ENERGY STAR dishwashers annually save you 5,000 gallons of water, $40 on your energy bill, and 230 hours (that you get to spend NOT washing dishes).

Hot Water Heaters set at 120°F will save energy without sacrificing comfort. You'll still have plenty of hot water. Check your water heater today and turn it down if it's above 120F. Some water heaters mark 120F with an arrow for the optimal setting.

Clothes Washer water temp set to warm or cold can save half the cost of washing a load of laundry – 90% of energy used for washing clothes is used heating the water. An ENERGY STAR washer saves about 20% of the energy and 35% of the water over other models.

LED Lighting can save $60/year by replacing 5 incandescent bulbs with LED bulbs. LEDs are more efficient and last 5 times longer than CFLs. You don't have to replace them all at once, start with the ones you use the most.

Refrigerators run more efficiently if kept full, set to 38F, and the seals are tight. Refrigerators and freezers are more efficient when full. Use water bottles to fill up extra space.

Stop Vampire Power! The average American household spends about $100 every year powering electronics when they're turned 'off'. Unplug power blocks when not in use. About 10% of home electricity costs are wasted on vampire power - that's all the electricity used by all of Italy in one year.

Rate from 1 to 7 how much you disagree or agree with each of the following statements.
The information provided here was useful to me.
The information provided here was new to me.

Quick Links were provided at the end of the survey with the same links provided in the "learn more" items in Energy Games.

2.0 Energy Games

Week 1: Small Changes, Big Savings!

Q1

Clue:	Has your house sprung a leak?
Question:	Which two low-cost investments can reduce cooling costs by 10% to 25%?
Answers:	a) Curtains and ceiling fans; b) New windows and skylights; c) Caulk and weather stripping; d) Popsicles (Two-sided only)
Insight:	Weatherizing your home is an easy and inexpensive DIY project that can cost as little as $5.

Q2

Clue:	A bright future for Texas!
Question:	Which of these energy technologies saw over 50% increase in installations this year?
Answers:	a) Solar; b) Heat pumps; c) Energy Star HVAC; d) Small wind mills
Insight:	A solar system is installed in the US every 2.5 minutes!

Q3

Clue:	What does a 6 degree difference really feel like?
Question:	Which thermostat setting can save the typical Texas household 15% on cooling costs?
Answers:	a) 72F; b) 76F; c) 78F; d) 90F
Insight:	You can save 6-8% off your electric bill for each degree you increase your AC thermostat setting.

Q4

Clue:	What could power Texas through a drought?
Question:	Which energy source uses the least amount of water for the energy produced?
Answers:	a) Coal; b) Solar; c) Nuclear; d) Gas
Insight:	Neither solar PV nor wind power use water to produce energy, which makes renewable energy a great option for Texas where water can be scarce.

Q5

Clue:	Comfortable, but not too hot!
Question:	What temperature setting on your water heater will save you money without sacrificing comfort?
Answers:	a) 90F; b) 100F; c) 120F; d) 140F
Insight:	Check your water heater today and turn it down if it's above 120F. Some water heaters mark 120F with an arrow for the optimal setting.

Q6

Clue:	Sit back, relax.
Question:	Which way to wash dishes saves the most money?
Answers:	a) Dishwasher; b) Hand wash; c) Use hot water; d) Garden Hose
Insight:	ENERGY STAR dishwashers annually save you 5,000 gallons of water, $40 on your energy bill, and 230 hours (that you get to spend NOT washing dishes).

Q7

Clue:	Solar for $0 down?
Question:	Which is larger, the cost of leasing a solar installation or the amount it saves on

your electric bill?

Answers: a) Monthly lease; b) Monthly savings; c) It's a wash; d) Wait, you can lease solar?

Insight: Many solar leases require no down payment, include free maintenance, and monthly lease payments will be covered by reduced electric bills with cash to spare.

Q8

Clue: The high cost of keeping cool.

Question: For the typical Texas household, 40% of the electric bill is spent on what?

Answers: a) Lighting; b) Heating and cooling; c) Cooking; d) Vacuuming

Insight: An AC Tune-up can save up to 30%. Big energy users are a great place to start saving.

Q9

Clue: A number you'll appreciate!

Question: How many years does it take the typical solar system in Texas to pay for itself?

Answers: a) 8 years; b) 16 years; c) 20 years; d) Keep counting

Insight: Solar panel prices have dropped almost 50% in the last 5 years and can produce electricity for over 30 years, making it quicker and easier than ever to get a good return on your investment.

Q10

Clue: Cool off!

Question: Which of the tips below can save you nearly half the cost of washing a load of laundry?

Answers: a) Keep colors separate; b) Use warm instead of hot water; c) Use extra detergent; d) Only buy socks of one color

Insight: 90% of energy used for washing clothes is used heating the water.

Q11

Clue: Keep the cold where it belongs.

Question: You can reduce the cost of running your refrigerator by...

Answers: a) keeping it full.; b) setting the temp to 39F.; c) checking the seals.; d) all of these

Insight: Refrigerators and freezers are more efficient when full. Use water bottles to fill up extra space.

Q12

Clue: Tickled green!

Question: How do Texas solar system owners feel about their decision to install?

Answers: a) Extremely satisfied; b) Satisfied; c) Dissatisfied; d) Extremely dissatisfied

Insight: On a recent survey, Texas solar owners were very happy they decided to install a solar PV system. You can ask some of them on the DFW Solar Tour on Saturday, October 3.

Q13

Clue: Does your AC have a speed?

Question: To cool down your house to 78F, will setting the thermostat to 70F cool it faster than setting it to 78F?

Answers: a) ; b) ; c) Yes; d) No

Insight: Like coming home to cool house? A programmable thermostat can start cooling your house before you get home.

Q14

Clue: There's sunshine in Texas everywhere you look...

Question: Do solar panels have to face south?

Answers: a) ; b) ; c) Yes; d) No

Insight: Installing solar panels on the south side of your home produces the most energy, but solar panels still produce a lot of energy facing W, SW, SE, E. Your solar installer can recommend the best design for your home.

Q15

Clue: When you're taking off for a trip Around the World…

Question: How high should you set the thermostat when you leave the house to save money on cooling in the summer?

Answers: a) 75F; b) 80F; c) 85F; d) 90F

Insight: A programmable thermostat makes it easy to set the temperature higher when you are away, and can cost as little as $20 at your local hardware store.

Week 2: Bigger Changes, Bigger Savings!

Q1

Clue: It's the label that means energy-efficient!

Question: If it's time to replace your HVAC, looking for which label could save $200 per year?

Answers: a) StarEnergy.jpg; b) StarTexas.png; c) Starfish.png; d) StarCartoon.png

Insight: Oversized air conditioners can leave your home humid. Size your AC right to keep humidity, costs, and energy use lower!

Q2

Clue: Why wait?

Question: What will happen to solar prices over the next 3 years?

Answers: a) Fluctuate wildly; b) Increase; c) Stay the same; d) Decrease

Insight: After steep price drops, prices have leveled out and are expected to be fairly flat over the next 3 years. With prices leveling off and declining incentives and rebates, now is a great time to invest in solar!

Q3

Clue: Big savings from the sun!

Question: What home installation saves the typical Texas homeowner $110 on their monthly electrical bill?

Answers: a) ENERGY STAR windows; b) Insulation; c) Solar panels; d) Hydroelectric dam

Insight: In a recent survey, Texas solar owners reported an average monthly electric bill savings of just over a $110 a month. You can talk to local solar owners on the DFW Solar Tour October 3!

Q4

Clue: If Benjamin Franklin saved...

Question: How much does the average American household spend every year powering electronics when they're turned 'off'?

Answers: a) 0; b) 25; c) 50; d) 100

Insight: Unplug power blocks when not in use. About 10% of home electricity costs are wasted on vampire power - that's all the electricity used by all of Italy in one year.

Q5

Clue: It's cooler in the shade.

Question: While producing energy for your home, how can solar panels also reduce your cooling bill?

Answers: a) By shading your roof; b) By reducing your stress; c) By producing 'cool' energy; d) By being blue

Insight: Solar panels absorb the sun's heat, providing shade and keeping your house cool.

Q6

Clue: It adds up quickly...

Question: How long would it take an energy smart clothes washer to save as much water as a person drinks in a lifetime?

Answers: a) 1 year; b) 15 years; c) 50 years; d) Never

Insight: Front loading washers save energy and water! High efficiency washers use up to 50% less water and energy than standard models.

Q7

Clue: A handful of reasons to use the fan...

Question: A ceiling fan can save money on cooling by making the room feel how much cooler?

Answers: a) 1 degree; b) 3 degrees; c) 5 degrees; d) 42 degrees

Insight: The trick with ceiling fans is to remember to turn them off when you leave the room.

Q8

Clue: The sky's the limit.

Question: On what type of roof can solar panels be installed?

Answers: a) RoofTile.png; b) RoofAsphalt.png; c) RoofMetal.png; d) RoofAllAbove.png

Insight: Solar panels can be installed on almost any type of roof and can also be ground mounted. Whatever type of roof you have, there's a solution for solar.

Q9

Clue: A generation of savings...

Question: How many years can solar panels keep producing more than 80% of full power?

Answers: a) 5 years; b) 15 years; c) 25 years; d) 100 years

Insight: After 25 years, solar panel output will decline slightly, but the panels will keep producing power. Most solar panels come with 25-30 year warranties to guarantee it.

Q10

Clue: A silver lining...

Question: On a cloudy day, can solar panels still produce energy?

Answers: a) ; b) ; c) Yes; d) No

Insight: On a cloudy day solar panels produce less energy, but they still produce. And your AC won't have to work as hard on cloudy days either. Don't worry, if you need more energy, the grid has you covered.

Q11

Clue: A shocking combination!

Question: Which of these can an ENERGY STAR clothes washer save?

Answers: a) electricity; b) water; c) both; d) neither

Insight: An ENERGY STAR washer saves at least 20% of the energy and 35% of the

water over other models.

Q12

Clue: What does 'photovoltaic' mean anyway?

Question: The Investment Tax Credit provides 30% off of what?

Answers: a) A solar thermal system; b) Rain water collection ; c) A solar PV system; d) A wind farm

Insight: In addition to the 30% Federal Investment Tax Credit (ITC), local utilities offer additional rebates and incentives. Click the learn more link to find yours. (PV: Photo = light, Voltaic = voltage)

Q13

Clue: Since prices are dropping on LEDs...

Question: How much money can you save per year by replacing 5 incandescent bulbs with LED bulbs?

Answers: a) $10; b) $20; c) $40; d) $60

Insight: LEDs are more efficient and last 5 times longer than CFLs saving you more over the long run. You don't have to replace them all at once, start with the ones you use the most.

Q14

Clue: Can you bill the electric company?

Question: What does your solar system do when it produces more energy than you use?

Answers: a) Turns all the lights on; b) Sends it to the grid; c) Shuts the system off; d) Signals the international space station

Insight: Many retail providers will pay you for the extra energy you produce, but you may have to do a little research. Check with your utility or Retail Electric Provider (REP).

Q15

Clue: Knowledge is (solar) power.

Question: How can neighbors who have already installed solar help you?

Answers: a) Share research; b) Give advice on solar installers; c)Share experience with installation; d) All of the above

Insight: You can't test drive a solar installation, but talking to the neighbors can provide a lot of valuable experience and insight that can help you decide.
