# Supplementary material for: Small Is Big: Interactive Trumps Passive Information in Breaking Information Barriers and Impacting Behavioral Antecedents
Source: PLoS One. 2017 Jan 18;12(1):e0169326. doi: 10.1371/journal.pone.0169326 (PMC5242502; doi:10.1371/journal.pone.0169326)
Supplement: S1 Memo — (PDF) [file pone.0169326.s004.pdf]

Sandia National Laboratories

Operated for the U.S. Department of Energy by
Sandia Corporation
Albuquerque, New Mexico 87185-1019

SNL HSB

date: April 21, 2015

to: Kiran Lakkaraju, MS 1327

Terry

from: Terry J. Reser, MS-1015
Human Studies Board Administrator

subject: **"A Pilot Study to Assess Solar Salient Beliefs" (SNL1520) is Exempt**

Sandia's Human Studies Board has reviewed the information submitted for this proposed activity and has determined that the activity does constitute human subject research, but is Exempt from 10 CFR 745 under section 101(b)(2):

(2) Research involving the use of educational tests (cognitive, diagnostic, aptitude, achievement), survey procedures, interview procedures or observation of public behavior, unless:

(i) Information obtained is recorded in such a manner that human subjects can be identified, directly or through identifiers linked to the subjects; and

(ii) any disclosure of the human subjects' responses outside the research could reasonably place the subjects at risk of criminal or civil liability or be damaging to the subjects' financial standing, employability, or reputation.

Enclosed you will also find the approved Consent Agreement, which contains an HSB approval stamp on each page indicating the expiration date of the approval. **Only unaltered copies of this approved form may be used to document consent from human subjects.**

Even though this study is currently exempt from federal law, DOE Order 443.1B and Sandia policy for protecting human subjects still apply, and you are required to understand and carry out your responsibilities as PI for protecting human subjects of research. These responsibilities, as well as those of the HSB, are described in detail in the *HSB Procedures Manual* (http://www.sandia.gov/health/hsb/HSBmanual.pdf).

Please note that this determination is based on the current scope and description of the proposed study. If the scope of work or proposed activities should change (as in your proposed second human study), you must notify the HSB immediately. Also, you need to notify the HSB annually from this date of the current status of this project until this study ends.

If you have any questions or concerns regarding this determination, please don't hesitate to contact me at 845-9171 or treser@sandia.gov.

cc: HSB file (SNL1520)
